# Increasing Uptake of Silica Nanoparticles with Electroporation: From Cellular Characterization to Potential Applications

**DOI:** 10.3390/ma12010179

**Published:** 2019-01-07

**Authors:** Erick Phonesouk, Séverine Lechevallier, Audrey Ferrand, Marie-Pierre Rols, Christine Bezombes, Marc Verelst, Muriel Golzio

**Affiliations:** 1 Institut de Pharmacologie et de Biologie Structurale—UMR 5089, 205 route de Narbonne, 31077 Toulouse CEDEX 04, France; Erick_31@hotmail.fr (E.P.); rols@ipbs.fr (M.-P.R.); 2 Chromalys SAS, 29 rue jeanne Marvig, 31400 Toulouse, France; severine.lechevallier@chromalys.fr (S.L.); marc.verelst@chromalys.fr (M.V.); 3 IRSD, Université de Toulouse, INSERM (U1220), INRA, ENVT, UPS, 31024 Toulouse CEDEX 3, France; audrey.ferrand@inserm.fr; 4 UMR1037 INSERM, Université de Toulouse, ERL5294 CNRS, 31100 Toulouse, France; christine.bezombes@inserm.fr

**Keywords:** tumor targeting, drug delivery, electroporation, silica nanoparticles fluorescence imaging, electric field

## Abstract

In the fields of biology and medicine, nanoproducts such as nanoparticles (NPs) are specifically interesting as theranostic tools, since they offer the double capacity to locally deliver active drugs and to image exactly where the product is delivered. Among the many described possibilities, silica nanoparticles (SiNPs) represent a good choice because of their ease of synthesis, the possibility of their vast functionalization, and their good biocompatibility. However, SiNPs’ passive cell internalization by endocytosis only distributes NPs into the cell cytoplasm and is unable to target the nucleus if SiNPs are larger than a few nanometers. In this study, we demonstrate that the cell penetration of SiNPs of 28–30 nm in diameter can be strongly enhanced using a physical method, called electroporation or electropermeabilization (EP). The uptake of fluorescently labelled silica nanoparticles was improved in two different cancer cell lines, namely, HCT-116 (human colon cancer) cells and RL (B-lymphoma) cells. First, we studied cells’ capability for the regular passive uptake of SiNPs in vitro. Then, we set EP parameters in order to induce a more efficient and rapid cell loading, also comprising the nuclear compartment, while preserving the cell viability. In the final approach, we performed in vivo experiments, and evidenced that the labeling was long-lasting, as confirmed by fluorescence imaging of labeled tumors, which enabled a 30-day follow-up. This kind of SiNPs delivery, achieved by EP, could be employed to load extensive amounts of active ingredients into the cell nucleus, and concomitantly allow the monitoring of the long-term fate of nanoparticles.

## 1. Introduction

Since the twentieth century, innovative aspects in the nanosciences have been growing. Indeed, in the fields of biology and medicine, nanoproducts such as nanoparticles (NPs) are of particular interest. They are specifically interesting as theranostic tools because they can offer the possibility to deliver locally and specifically active drugs (and thus act as a therapeutic tool) while having the ability to image anatomical or pathological structures (and therefore also serve as diagnostic tools). This double capacity of NPs makes them promising devices, particularly useful in the management of many pathologies such as cancers, whose current therapies are frequently limited [1].

Besides their obvious interest, toxicity generated by metallic nanoparticles such as gold, iron, or silver [1,2,3,4,5] has been reported and might sometimes be of concern for therapeutic applications. Silica NPs (SiNPs) represent a nanomaterial of choice, because of their ease of synthesis [6]; their possibility of being covalently functionalized with fluorescent moieties; their biocompatibility (as evidenced in murine models) [7]; their biodistribution [8]; and their biodegradability in vivo, as tissues degrade silica to orthosilicic acid [9], which is eliminated from the body. These characteristics make silica a perfect material for biomedical use, and SiNPs have been used for a number of pharmaceutical applications [10,11,12,13,14,15,16,17].

As mentioned above, SiNPs are biofunctionalizable [18], which means their surface can be chemically modified, giving them additional properties such as interaction specificities, better stabilization against aggregation, or imaging properties. For example, the functionalization of NPs with folic acid confers them the property to specifically target tumor cells [7], while polyethylene glycol (PEG) coating allows NPs to persist in the bloodstream [8] or be invisible to the immune system, preventing their opsonization by macrophages [19]. Moreover, synthesis procedures allow the incorporation of fluorophores or contrast agents within the nanoparticle matrix during the NPs’ synthesis protocols, making fluorophores an integral part of the nanostructure [20], and thus resulting in a completely traceable NP, both in vitro or in vivo.

The mechanism of the uptake of silica-based NPs by cells appears to be mediated by an active endocytosis process. Indeed, cellular uptake is inhibited by a decrease in temperature to 4 °C and incubation with metabolic inhibitors [21]. The endocytosis of SiNPs may also vary from one cell type to another, and might be difficult in certain cases. Moreover, NPs’ internalization by cells following endocytosis pathways only distributes NPs inside the cell cytoplasm, and is unable to target the nucleus if NPs are bigger than few nanometers [22], which may represent a problem for efficient drug delivery [23]. However, SiNPs’ cell penetration can be enhanced by a physical method called electroporation or electropermeabilization (EP) [24,25].

EP consists of the application of electric field pulses to cells, which causes a transient permeabilization of the cellular plasma membrane and increases the intracellular passage of hydrophilic molecules [26,27,28]. EP allows the precise targeting, and thus treatment, of the tissue (or cells) in between the electrodes [29]. In addition to its ease and speed of application, this technique is inexpensive and has low toxicity. These advantages currently allow this method to undergo a significant expansion, and EP is increasingly being used in clinics [30,31]. 

The parameters of the electrical pulses applied are the voltage (in volts), the duration (in micro or milliseconds), the frequency (in Hertz), and the number of pulses [32]. Electrical parameters are chosen according to the type of molecule that has to be transferred. Since nucleic acids are large and charged macromolecules, they cannot enter cells by diffusion across the plasma membrane. The optimized electric field parameters are called electrogenotherapy (EGT) parameters. In EGT, long-lasting electrical pulses are usually preferred to allow the electrophoretic forces to push nucleic acids into the cells during the electric field application [28,33]. When the molecule to be transferred is a small hydrophilic molecule such as bleomycin, which is used in electrochemotherapy (ECT), its entry into cells mainly occurs by diffusion via transient permeabilization structures within cell membranes [34]. Thus, the application of a calibrated electric field to on cells (in vitro) or tissues (in vivo) allows the free access of several types of molecules into the cytoplasm or nucleus of cells. However, the molecular mechanisms are still unknown, and the permeabilization structures (or membrane pores) are poorly characterized. Kim, et al. [24] performed a quantitative screening and direct visualization of uptake directionality for a set of fluorescent molecules and fluorescence-doped NPs using electric-pulsation. They observed that the uptake intensities of fluorescent-doped NPs depended upon their sizes and the external electric voltages applied. Their studies suggest that nanoparticle entry pathways may be different depending on NPs’ composition, size, and fate in the cell. 

Taken together, NPs’ clinical use is still limited, and could potentially be improved by increasing the penetration efficiency into the cells of interest. Namely, in the absence of an electric field, endocytic pathways were described, but no access to the nucleus was observed [35,36].

In this study, we aimed to improve fluorescent SiNPs’ uptake in two different cancer lines: HCT116 (human colon cancer) cells and RL (follicular lymphoma) cells. We used two types of fluorescent SiNPs provided by the ChromaLys Company. The first one, called LumiLys 650, embeds a ruthenium fluorescent complex, which is well-adapted for in vitro microscopy in the visible light range (λ_ex_ = 365–500 nm; λ_em_ = 650 nm). The second one encapsulates a Cy7 dye, which is specially dedicated to in vivo imaging in the near-infrared (NIR) range (λ_ex_ = 750 nm; λ_em_ = 780 nm). First, we studied the cell lines’ capability for natural SiNPs uptake in vitro. Subsequently, we set EP parameters in order to increase SiNPs’ uptake without affecting cell viability. After successful and significant in vitro transfer of SiNPs to cells, we performed in vivo experiments to study the labeling stability. Future perspectives of the use of these new SiNPs were finally explored for the diagnosis or treatment of tumors.

## 2. Results

### 2.1. SiNPs Characterizations

**Figure 1:**
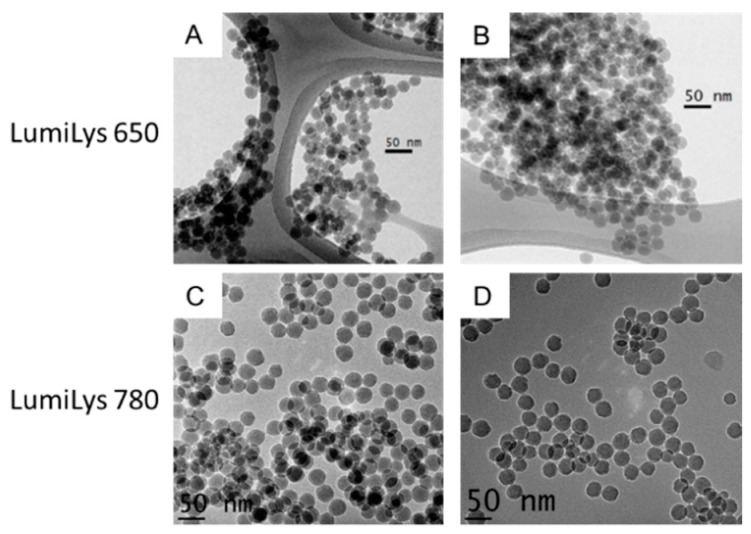
Physical characterization of LumiLys silica nanoparticles (SiNPs). Transmission electron microscopy micrographs of (**A**,**B**) LumiLys 650 NPs functionalized with Gd-DTPA (Gadolinium- DiethyleneTriaminePentaacetic Acid) and (**C**,**D**) LumiLys 780 NPs.

SiNPs (LumiLys 650 and LumiLys 780) were visualized by transmission electron microscopy (TEM) and appeared spherical and mono-dispersed with an average diameter of 28 nm (±2 nm) for LumiLys 650 and 30 nm (±2 nm) for LumiLys 780 (Figure 1), which was confirmed by dynamic light scattering (DLS) analyses (Figure S1). 

Ruthenium complex and Cy7 content were optimized in order to maximize the brilliancy of NPs. When too diluted, the brilliancy was weaker due to the small number of encapsulated chromophores, but embedding too many fluorophore molecules also led to a drastic brilliancy decrease caused by confinement and the self-quenching effect (Figure S2A).

Photoluminescence properties of both SiNPs types were studied in water at a concentration of 1 mg/mL. The photo-luminescence (PL) spectra of LumiLys 650 NPs showed a broad emission band at 650 nm and two broad excitation bands centered at 365 nm and 488 nm, respectively (Figure S3A). Consequently, this compound was efficiently excited in a broad wavelength range from 350 to 530 nm. In addition, emission intensity was monitored continuously over a 6 h period of time (Figure S2B). The emission intensity did not vary significantly during this period of time, indicating that the encapsulation of the dye inside these SiNPs allowed a good protection against photo-bleaching. In LumiLys 780 NPs, PL spectra showed a broad emission band at 780 nm and a broad excitation band centered at 750 nm (Figure S3B). As excitation and emission maxima are located in the NIR range, corresponding to the transparency window for biological tissues, they are particularly well-adapted for in vivo experimentation. Moreover, LumiLys 780 NPs were found not sensible to photo-bleaching.

### 2.2. Passive Incorporation of LumiLys 650 NPs into Cancer Cell Lines

#### 2.2.1. Effect of LumiLys 650 NPs Concentrations on Cancer Cell Viability

**Figure 2:**
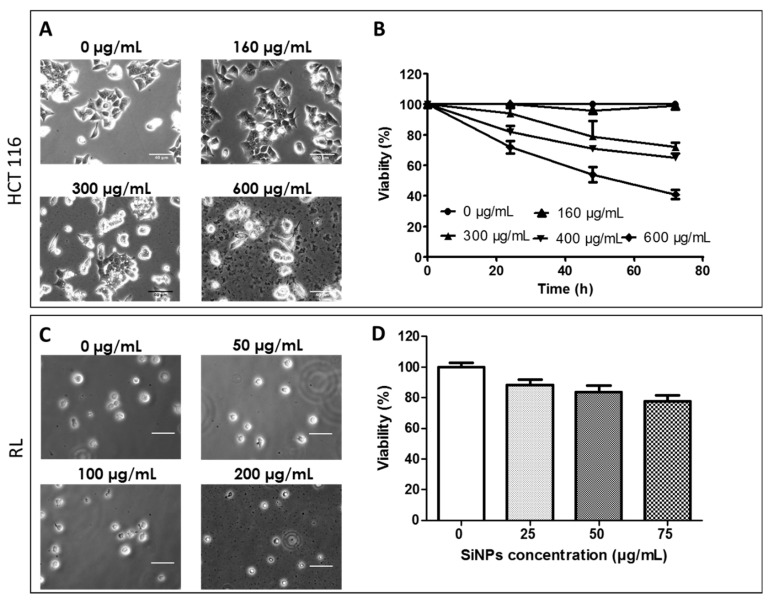
Comparison of cell proliferation in presence of increasing LumiLys 650 NPs concentrations. HCT-116 and RL cells were incubated in the presence of increasing concentrations of LumiLys 650. (**A**,**C**) Contrast phase micrographs of (**A**) HCT-116 and (**C**) RL cells as a function of SiNPs concentration at 24 h. (**B**,**D**) Percentage of viability for various concentrations of SiNPs for (**B**) HCT116 as a function of time and (**D**) RL cells 24 h after incubation. Histograms represent the mean +/− S.D. of three independent experiments.

Toxicity of SiNPs on HCT-116 and RL cells was determined by measuring cell viability after the passive incorporation of increasing SiNP concentrations. For HCT-116, no toxicity was observed below 160 μg/mL. In the case of LumiLys 650 NPs at concentrations up to 160 μg/mL, more than 90% of the cells remained alive (Figure 2A,B). The viability reached 75% at 300 μg/mL and became less than 50% at 600 μg/mL, 72 h after incubation. Thus, a SiNPs concentration of 160 μg/mL was used for further experiments. In RL cells, a more drastic effect of LumiLys 650 NPs was observed. Cell viability became less than 60% at 100 μg/mL 24 h after incubation (data not shown). The viability reached 80% at 75 μg/mL 24 h after incubation (Figure 2C,D). Thus, a SiNPs concentration of 75 μg/mL was chosen for further studies. 

#### 2.2.2. LumiLys 650 NPs Incorporation Kinetics into HCT-116 and RL Cells

**Figure 3:**
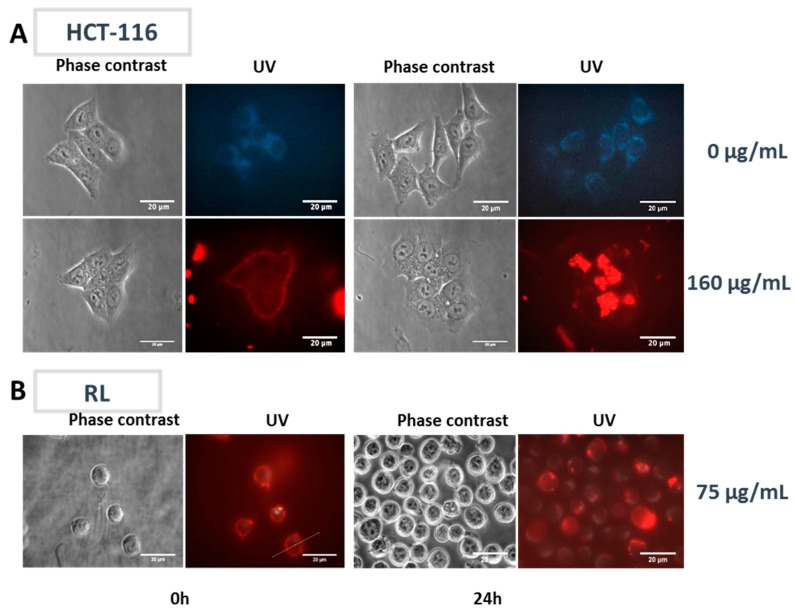
Visualization of HCT-116 and RL cells in the presence of LumiLys 650 NPs. HCT-116 and RL cells were incubated with or without LumiLys 650 NPs in the culture medium and visualized by wide field fluorescence microscopy (63× magnification). After 24 h of incubation, cells were first imaged by phase contrast and then by UV excitation using a UV filter (λ_exc_: 340–380 nm, λ_em_: 425 nm long-pass filter). Under UV excitation, cell autofluorescence appeared in blue while SiNPs’ fluorescence appeared in red. (**A**) HCT-116 were incubated with or without LumiLys 650 NPs (at 160 μg/mL) and (**B**) RL cells (at 75 µg/mL) in the culture medium.

HCT-116 and RL cells were incubated with or without LumiLys 650 NPs in the culture medium and observed by fluorescence microscopy (Figure 3). At t0, a slight adsorption of the LumiLys 650 NPs to the plasma membrane and the presence of aggregates outside the cells were observed. Over time, LumiLys 650 NPs accumulated inside the cells. After 24 h, LumiLys 650 NPs were clearly observed in the cell cytoplasm of both cell lines. 

The results obtained both on HCT-116 and RL cells showed that LumiLys 650 NPs interacted with the plasma membrane of cells in the first minutes of incubation and were able to enter passively into the cytoplasm without reaching the nucleus. LumiLys 650 NPs intensity signals in RL cells were lower in comparison with those observed in HCT-116 cells. The labeling was stable but decreased as a function of time upon cell division (data not shown).

#### 2.2.3. Subcellular NP Localization in Cancer Cell Lines

**Figure 4:**
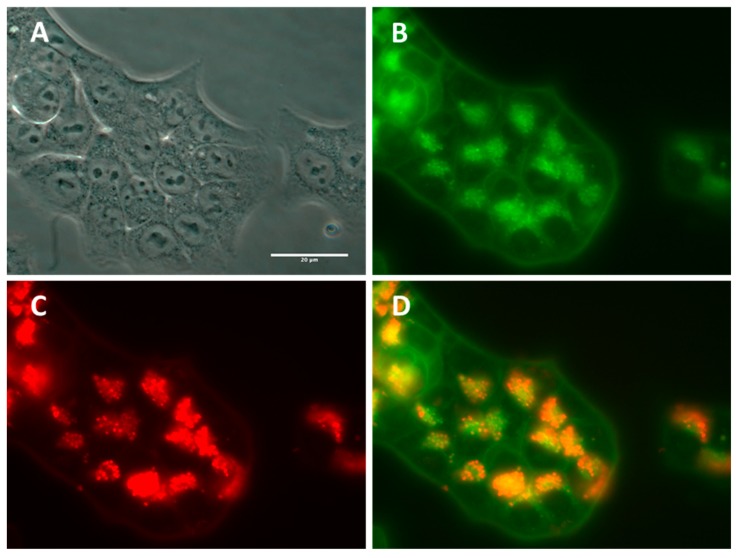
Lysosomal localization of LumiLys 650 NPs in HCT-116 cells. HCT-116 cells were incubated with the LumiLys 650 NPs (100 μg/mL) for 48 h, and lysosomes were labeled with a commercial Lysotracker^®^ lysosome tracking kit. (**A**) Phase contrast and (**B**) green fluorescence images of Lysotracker were obtained at 63× magnification. (**C**) LumiLys 650 NPs appear in red. (**D**) Merge channels showed co-localization of both signals in yellow. Scale bar corresponds to 20 µm.

In order to determine the subcellular localization of SiNPs after passive loading, lysosomes of HCT-116 cells were labelled with a commercial Lysotracker^®^ kit. HCT-116 cells were incubated with the LumiLys 650 NPs (100 μg/mL) for 48 h, and lysosomes were labeled. As shown in Figure 4, a co-labelling between lysosomes and LumiLys 650 NPs was observed by fluorescence microscopy, indicating that LumiLys 650 NPs accumulated in lysosomes over time following a perinuclear localization. As extensively reported by other authors [21], this specific NPs localization relied on a mechanism of passive incorporation by endocytosis. Of note, no SiNPs penetrated the nucleus.

### 2.3. Effect of Electric Field Application on SiNPs’ Incorporation into Cancer Cells

#### 2.3.1. Determination of Electrical Parameters for HCT-116 and RL Cells

**Figure 5:**
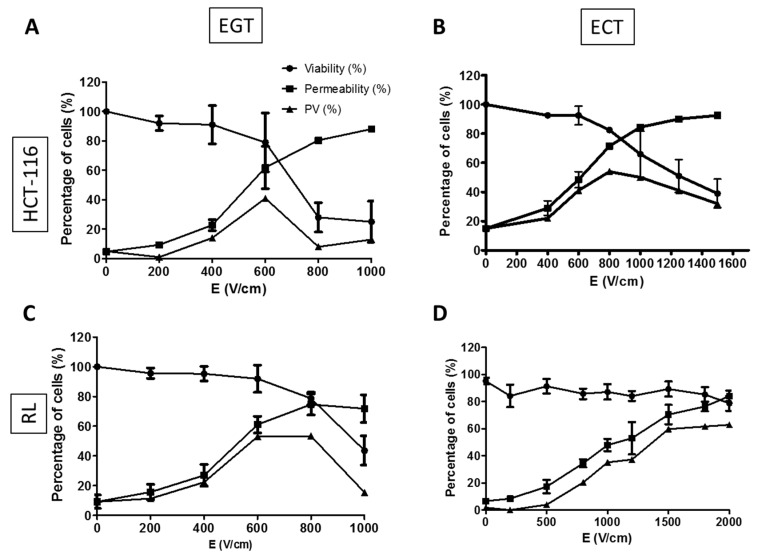
Percentages of viability, permeabilization, and permeable/viable cells as a function of the electric field intensity. (**A**,**C**) Electrogenotherapy (EGT) parameters: 10 pulses lasting 5 ms at 1 Hz frequency were applied to (**A**) HCT-116 and (**C**) RL cells in suspension. (**B**,**D**) Electrochemotherapy (ECT) parameters: 8 pulses lasting 100 µs at 1 Hz frequency were applied to (**B**) HCT-116 and (**D**) RL cells in suspension. Viability (round symbol) and permeability curves (square symbol) as a function of electric field (E) amplitude are plotted on each graph. The permeable and viable cells curve (triangle symbol) results from Equation (1). Graphs represent the mean +/− S.D. of three independent experiments.

EP is known to facilitate molecule uptake [27,28,33,37] by cells. We first performed an experiment using EGT (electrogenotherapy) parameters that induced a higher macromolecule uptake than ECT (electrochemotherapy) parameters [30,31,38,39]. Each cell line has specific characteristics, and thus are more or less sensitive to electric pulses. It was therefore necessary to determine the optimal amplitude of electric field E (V/cm) to use on the HCT-116 and RL cell lines. Permeability and viability tests were performed using either 10 pulses of 5 ms (EGT parameters) or 8 pulses of 100 µs (ECT parameters) at 1 Hz frequency on HCT-116 and RL cells suspended in HEPES buffer (Figure 5). 

From Figure 5A, the percentage of permeabilized HCT-116 cells was proportional to the increase of the electric field, and inversely proportional to cell viability. With EGT parameters, a viability higher than 50% was observed between 0 and 600 V/cm, whereas the permeability was higher than 50% between 600 and 1000 V/cm. The curve resulting from the equation giving the percentage of permeable and viable cells showed a peak at 600 V/cm corresponding to a maximum of 40% of viable permeabilized cells for HCT-116 cells EGT electropermeabilization in suspension. From Figure 5B, 800 V/cm was selected for HCT-116 for ECT parameters, corresponding to 50% of viable permeabilized cells.

The same experiments were performed with RL cells using EGT (Figure 5C) and ECT (Figure 5D) parameters. The selected values were 700 V/cm for EGT parameters, corresponding to a maximum of 50% of viable permeabilized RL cells, and 1500 V/cm for ECT parameters, corresponding to a maximum of 60% of viable permeabilized RL cells. 

The difference of the electric field amplitude between HCT-116 and RL cells was in agreement with the fact that the size of the RL cells was smaller than the size of HCT-116 cells in suspension [37].

#### 2.3.2. SiNPs Intracellular Localization after Cancer Cell Electropermeabilization

**Figure 6:**
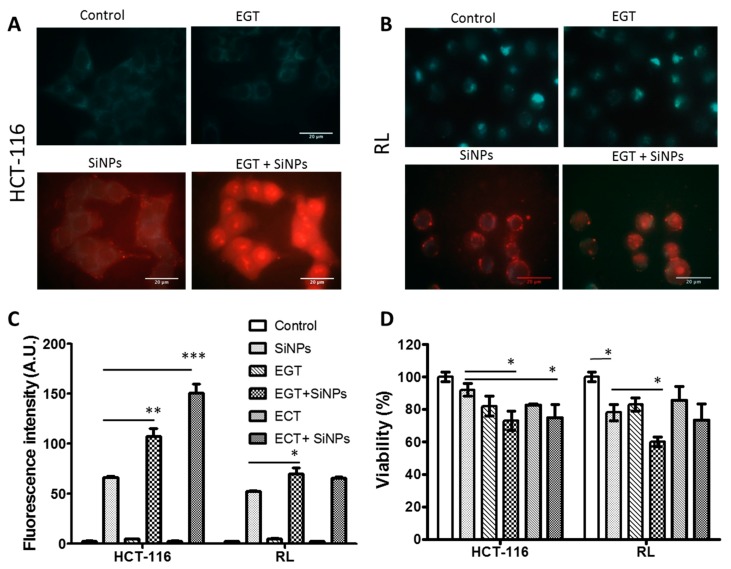
LumiLys 650 NPs uptake in cells and viability upon electropermeabilization. (**A**,**B**) Visualization of the electrotransfer of LumiLys 650 NPs after EGT (10 pulses 5 ms, 700 V/cm, 1 Hz) in (**A**) HCT-116 and (**B**) RL cells. (**C**) Histogram representing the average fluorescence intensity of HCT-116 and RL cells 24 h after treatment. For HCT-116 cells, EGT (10 pulses 5 ms, 700 V/cm, 1 Hz) and ECT (8 pulses 100 µs, 800 V/cm) parameters were applied in the presence of 100 μg/mL SiNPs. For RL cells, EGT (10 pulses 5 ms, 700 V/cm, 1 Hz) and ECT (8 pulses 100 µs, 1500 V/cm) parameters were applied in the presence of 75 μg/mL LumiLys 650 NPs. (**D**) Histogram representing the average HCT-116 and RL cell viability 24 h after treatment for the corresponding experimental conditions. Histograms represent the mean +/− S.D. of three independent experiments. Data were considered statistically significant from a p-threshold of less than 0.05 (* *p* < 0.05; ** *p* < 0.01; *** *p* < 0.001).

Microscopic observation allowed us to directly observe the transfer of LumiLys 650 NPs into the cells following the application of PEF (Pulsed Electric Field). Figure 6A,B shows fluorescence micrographs of HCT-116 and RL cells before and after EP. The fluorescence signal increased after EP, suggesting that LumiLys 650 NPs entered into the cells immediately after EP. Importantly, both cytoplasm and nucleus were labeled (Figure 6A,B), indicating that LumiLys 650 NPs not only penetrated into the cytoplasm, but could also reach the nucleus; while in absence of EP, SiNPs’ uptake was observed only in the lysosomes (Figure 4).

Quantitative analysis of SiNPs incorporated into the cells was determined by measuring the fluorescence intensity by flow cytometry 24 h after EP, as shown in Figure 6C. For HCT-116 cells, experimental conditions without LumiLys 650 NPs (control and ECT or EGT) displayed a weak fluorescence intensity. When LumiLys 650 NPs were incubated 24 h with cells (SiNPs), the fluorescence intensity was much higher. EP enhanced LumiLys 650 NPs uptake, as the mean fluorescence intensity showed a significant increase in comparison to LumiLys 650 NPs alone. Indeed, a 1.5-fold and a 2-fold increase were observed when EGT and ECT parameters were applied, respectively. Similar results were observed with RL cells with a 1.5-fold increase when EGT and ECT parameters were applied. 

Considering SiNPs incorporation-associated cell viability, we observed that both EGT and ECT treatments alone slightly affected the viability of the HCT-116 and RL cells (80% viability). In the presence of SiNPs, the two cell lines displayed different behavior. Indeed, while EGT and ECT parameters induced a small decrease in the viability of HCT-116 cells, RL cells were less affected by ECT than EGT parameters. 

Altogether, these results showed that ECT parameters gave a better SiNPs uptake/viability ratio. Indeed, a better SiNPs uptake was shown with an equivalent viability for HCT-116 cells as when treated by EGT, while a comparable SiNPs uptake with a better viability occurred for RL cells. Therefore, EP represents a rapid and efficient way to transfer SiNPs in various cell lines while preserving their viability. This kind of cell labeling prior cell (re)injection in vivo could be important in advanced therapy medicinal products, such as in somatic-cell therapy medicines, where cells or tissues are taken from donors, subsequently manipulated/altered, and introduced into the patient to cure, diagnose, or prevent disease. 

#### 2.3.3. Effect of Electropermeabilization on SiNPs Trafficking into Cells

**Figure 7:**
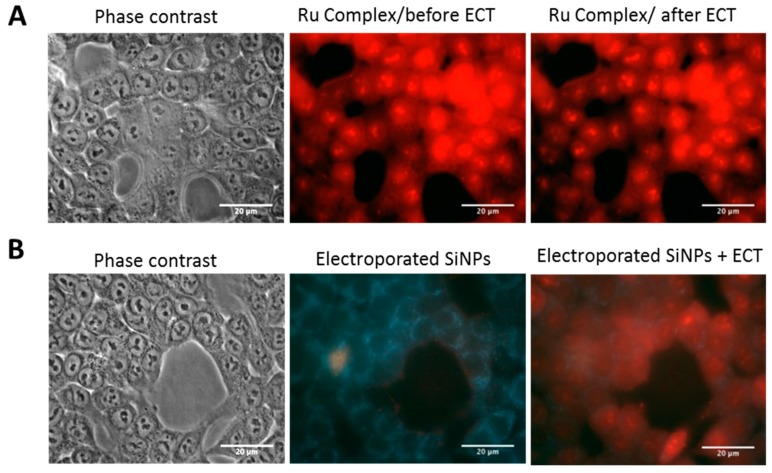
Visualization of cell labelling in HCT-116 cells. HCT-116 cells were incubated with (**A**) Ruthenium complex alone and (**B**) electroporated LumiLys 650 NPs and then visualized by wide field fluorescence microscopy (63× magnification). Phase contrast (first column) and fluorescence observations were performed 10 min after incubation with SiNPs before EP (second column) and just after ECT parameters (8 pulses lasting 100 µs at 700 V/cm) (third column). Under UV excitation (λ_exc_: 340–380 nm, λ_em_: 425 long-pass filter), cell autofluorescence appeared in blue while SiNPs’ fluorescence appeared in red. Scale bars correspond to 20 µm.

In order to address the question of whether LumiLys 650 NPs were sensitive to EP itself, we designed an additional set of experiments. Indeed, SiNPs subjected to an electric field could disintegrate and thus release the ruthenium (Ru) complex. When the Ru complex alone was incubated with cells, we observed that the Ru complex was by itself capable of entering the cell in a few seconds, even before the application of the PEFs with ECT parameters (Figure 7A). In order to test the Ru complex release hypothesis, a LumiLys 650 NPs suspension alone was subjected to an electric field of 800 V/cm as it was applied to cells. The SiNPs suspension was then deposited on HCT-116 cells and immediately observed by fluorescence microscopy (Figure 7B). Over 10 min, no signal was observed inside the cell, and LumiLys 650 NPs were only adsorbed to the outer membrane of the cell, proving that SiNPs were not affected by the electric field and did not release any Ru complex (Figure 7B). When the same cells were then electropermeabilized, LumiLys 650 NPs entered quickly into HCT-116 cells (Figure 7B). The same experiments were performed with RL cells (data not shown). These experiments clearly indicated that LumiLys 650 NPs remained intact after being exposed to electric field pulses. 

A time lapse acquisition was performed on HCT-116 and RL cells in the presence of the LumiLys 650 NPs and pulsed directly under the microscope. In videos, we could see a uniform cell labelling (see Supplementary Data videos 1 and 2). No asymmetry was observed, as was already described for nucleic acids or propidium iodide [28,33]. 

### 2.4. In Vivo Monitoring of LumiLys 780 NPs Labelled Cells

EP represents an original way of concentrating SiNPs inside tumor cells for new vectors development, for diagnosis or treatment in cancer therapies. In order to see if labelled cells could be detected in vivo by small animal optical imaging, SiNPs-labeled RL cells were grafted into immunodeficient mice. For these experiments, LumiLys 650 NPs were replaced by LumiLys 780 NPs, which are more suitable for in vivo imaging. Additional questions were addressed: are labelled cells able to develop tumors? How long could SiNPs efficiently label a tumor in vivo without being destroyed, metabolized, or excreted? Finally, can SiNPs-labeled cells be used for longitudinal tumor detection? 

**Figure 8:**
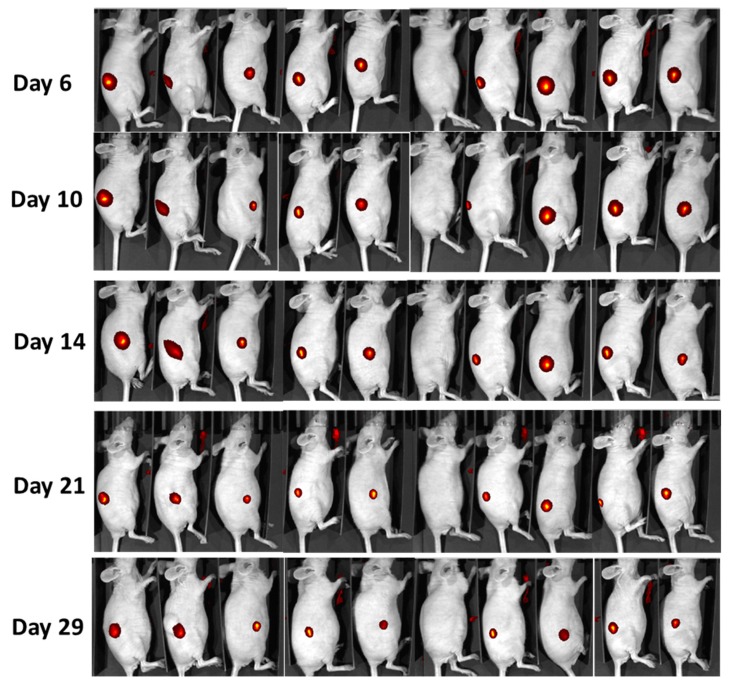
In vivo tumor monitoring by fluorescence macroscopy. For this, 2.5 × 10^6^ RL Cy7-labeled cells were injected subcutaneously (sc) into the right flanks of Nude mice (n = 10). Tumor growth was followed by in vivo imaging fluorescence (λ_ex_ = 750 nm; λ_em_ = 780 nm). Images show each individual mouse at different time points.

EP was used for ex vivo rapid cell loading by LumiLys 780 NPs on RL tumor cells. Then, labeled RL cells were subcutaneously injected into the flank of the nude and SCID mice. Tumor detection was assessed by small animal optical imaging with IVIS spectrum for visualization of SiNPs fluorescence (Figure 8). 

The monitored tumor growth showed that 90% of nude mice (N = 10) developed a tumor. The tumor growth follow-up using fluorescence emission of labelled cells was possible in the NIR range for at least 1 month (Figure 8). Clearly, cell labeling with SiNPs did not impair tumor growth behavior.

## 3. Discussion

The results presented in this study provide evidence that the EP of SiNPs allowed a better and more efficient cell loading, and a rapid entry into the cell with penetration to the nucleus while preserving the cell viability. This labeling was long-lasting, as demonstrated with in vivo fluorescence imaging of labeled tumors over a period of one month.

The in vitro labeling of cells with SiNPs by an endocytotic process led to efficient uptake and remained acceptable since cell behavior was not altered. Yet, in some cell lines (i.e., RL), the SiNPs endocytosis process was not efficient, and did not lead to a satisfactory NP internalization rate. Nevertheless, EP could be favorable in these cases, and could drastically increase NP loading into the cells, provided that the optimal electrical parameters are applied, to maintain cell viability even for important cell loading. Here, we showed that ECT parameters (already used in clinics) could enhance SiNPs uptake, while preserving the viability of the cells.

Moreover, a rapid entry of SiNPs was observed upon application of EP. This could be of relevant importance when cells are injected in vivo or when very sensitive cell lines (e.g., primary cells) are concerned, and thus low NP concentration labeling conditions are favored. Indeed, we showed that EP allowed rapid ex vivo cell loading, thus accelerating the cell labeling process. This result is very important for in vivo applications, when specific labeling is envisaged in organs other than the liver and the spleen. Generally, NPs are promptly captured by the reticuloendothelial system (mainly local macrophages, which engulf NPs in a few minutes to a few hours), and are stored in the spleen and liver [23]. Yet, the application of different techniques that could allow a specific homing of nanoparticles to zones located elsewhere in the body would represent an additional therapeutic advance. Indeed, nanoparticle functionalization has often been proposed to bypass nanoparticle sequestration by the liver and spleen, but surface ligands are often stripped off nanoparticles, or covered by opsonins. Thus, nanoparticles capture by the liver and spleen is generally only a matter of time. Physical means to deliver nanoparticles elsewhere are thus required, and would surely complement the chemical means for a targeted delivery. In such cases, we could achieve an in vivo cell labeling procedure, which would be governed by a very fast internalization technology and could bypass the standard clearance paths. Surely, EP represents a solution of choice for this kind of application, when specific design of the set of electrodes is used to target the cancerous tissue [40]. Another important observation of this study was the penetration of the SiNPs into the nucleus of the cell. As already mentioned, nucleus-targeted drug delivery is a promising strategy for anticancer therapy, and in vivo nucleus targeting using SiNPs as Trojan horses would be very challenging. Limited by the channel size of the nuclear pores, vehicles that enter the nucleus via the nucleopore should be very small and decorated with a nuclear localization signal (NLS) [41]. However, the tiny size may promote leakage of vehicles or a rapid renal clearance, and the positively charged NLS can lead to strong non-specific interactions in vivo. In the present study, we demonstrate that cell EP should be a very interesting alternative for in vivo nucleus targeting.

According to the literature, the entry of small exogenous molecules such as propidium iodide is asymmetric, mainly because the permeabilized zones of the cell are more important on the anode-facing side [33]. For larger macromolecules such as nucleic acids (i.e., DNA, SiRNA), electrophoretic processes are involved, pushing the negatively charged molecules toward the cathode [28]. The entry of SiNPs into cells during EP did not evidence this asymmetry, as they were positively charged (see videos 1 and 2). We therefore concluded that LumiLys 650 NPs entry was achieved by passive diffusion following EP and not by electrophoresis. This was already proposed by Kim and coworkers [24,25].

The entry of LumiLys 650 NPs into the nucleus indicated that the size of the nuclear pores was large enough to allow the passage of the SiNPs. Interestingly, Bellard and Teissié [42] showed that the application of EP can induce a 70% increase in cell nucleus size and can potentially increase the size of nuclear pores. Therefore, under EP, after diffusion across the permeabilized plasma membrane, the 28 nm LumiLys 650 NPs could easily cross the nuclear barrier. This is another very important result of this work, and this property could be widely exploited in therapeutic applications where the vectorization of a drug acting directly on nuclear DNA is required. However, the mechanism involved remains to be fully elucidated, and several questions still remain: is the entry of NPs by EP dependent on NPs size? Beyond a certain NPs size, is the process inhibited? Is the surface charge of NPs an important parameter? Further work needs to be done to answer these important questions.

Finally, we showed that SiNPs labeling of cells by EP allowed tumor cell tracking over 30 days, as demonstrated with in vivo fluorescence imaging, and that tumor growth was not impaired. This was due to one of the important characteristics of SiNPs, which is their excellent resistance to metabolic degradation, thus allowing a long-lasting cell labeling. Coupled with the above-mentioned characteristics of EP, one can thus hope for very beneficial uses of this technology in many theranostic applications. However, before being able to effectively use all the above-mentioned advantages of EP in vivo, a certain number of questions will have to be explored to determine if this technology makes it possible to precisely quantify the tumor size as well as the processes of tumor progression or regression, associated with the administration of a treatment, for instance.

## 4. Materials and Methods

### 4.1. Materials

**Figure 9:**
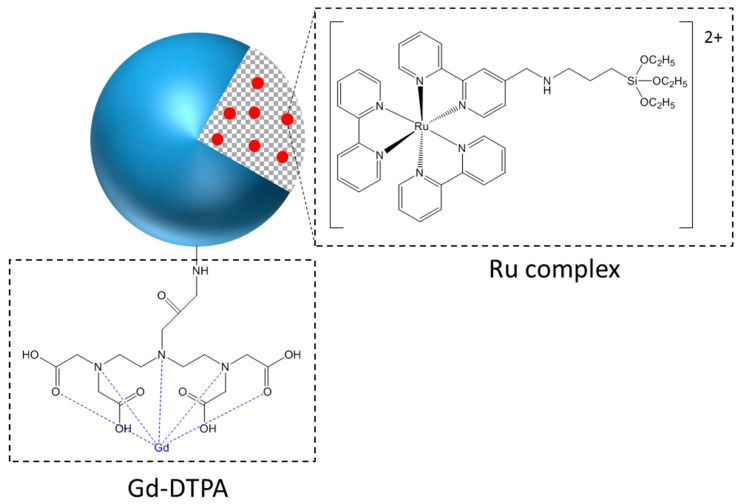
Schematic representation of a LumiLys 650 NP functionalized with Gd-DTPA (Gadolinium- DiethyleneTriaminePentaacetic Acid).

Acetonitrile, 3-aminopropyltriethoxysilane (APTES), diethylenetriaminepentaacetic acid (DTPA), gadolinium chloride hexahydrate, and dimethylsulfoxide (DMSO) were purchased from Sigma Aldrich (St Quentin Fallavier, France). Acetic acid was obtained from Fluka. Ethanol was obtained from Panreac. All SiNPs were provided by Chromalys (Toulouse, France). They consisted of 25 nm diameter SiNPs, incorporating different fluorophores. LumiLys 780 NPs incorporated the cyanine 7 fluorophore, which confers a near-infrared fluorescence to the SiNPs, with an emission centered on 780 nm under 750 nm excitation. This wavelength range is well-adapted for in vivo experiments. LumiLys 650 NPs incorporated a silylated ruthenium tris-bipyridine complex (Figure 1), which confers a red fluorescence to the nanoparticles, centered at 648 nm under 365–500 nm excitation (two maxima at 365 and 488 nm). For a better dispersion in water, LumiLys NPs used for experiments were functionalized with Gd-DTPA (Gadolinium- DiethyleneTriaminePentaacetic Acid) (Figure 9). Moreover, this paramagnetic functionalization allows the use of these particles in magnetic resonance imaging (MRI), which could be very useful in the future.

### 4.2. Grafting of Gd-DTPA

Gd-DTPA grafting on LumiLys NPs was described by Lechevallier et al. [43]. This grafting plays two roles: (i) improves SiNPs’ stability in aqueous media and (ii) gives in vivo MRI detection capability (not used in this study, but useful for further in vivo investigations). Briefly, 50 mg of LumiLys NPs were suspended by sonication in 15 mL of acetonitrile. Two hundred and fifty microliters of APTES were added dropwise, and the suspension was agitated at 50 °C for 24 h. The NPs were recovered by centrifugation (4000 g for 10 min) and washed three times in ethanol. Then, amine-modified nanoparticles were suspended in 12 mL of a mixture ethanol/acetic acid (1/1). The DTPA (15 mg) was added and the suspension was aged under reflux at 85 °C overnight. Nanoparticles were collected by centrifugation (4000 g for 10 min), washed three times with an acetone/acetic acid mixture (1/1), and washed three more times with water to remove any unreacted DTPA molecules. The purified nanoparticles were re-suspended in 10 mL of water, and 13.35 mg of Gd chloride was added. A limpid suspension was obtained while shaking at room temperature for 24 h (and purified by centrifugation: 17,000 g for 1 h). Zeta potentials of as-functionalized SiNPs were measured at +17 mV (in water) for both SiNPs types.

### 4.3. Instrumentation

Particle shape and size were examined via transmission electron microscopy (TEM), using a Philips Model CM20 microscope (Eindhoven, The Netherlands). Size distribution was assayed by measuring the size of 200 NPs using Image J software. Size and zeta potential measurements were analyzed by dynamic light scattering (DLS) using a Zetasizer Nano (Malvern instruments, Malvern, UK). The luminescence of samples in suspension at a water concentration of 1 mg/mL was studied with a Jobin-Yvon Model Fluorolog FL3-22 spectrometer (HORIBA Scientific, Montpellier, France) equipped with a R928 Hamamatsu photomultiplier and a 450-W Xe excitation lamp. UV-visible absorption spectrum for Ru complex concentration evaluation was performed on a Varian Cary 5000 (Agilent Technologies, Santa Clara, CA, USA).

### 4.4. Cell Culture, Cytotoxicity Tests, and Fluorescence Imaging

Adherent Human Colorectal Carcinoma (HCT-116) cells from the American Type Culture Collection (ATCC, Rockville, MD, USA) were cultivated in DMEM medium containing 4.5 g/L d-glucose, 1% antibiotic (penicillin/streptomycin) and supplemented with 10% decomplemented fetal bovine serum (FBS), and maintained at 37 °C under 5% CO_2_. The HCT-116 cells in suspension were collected after trypsin treatment. The human follicular lymphoma cell line RL obtained from the ATCC was cultured in suspension in complete RPMI 1640 medium at 37 °C in a humidified atmosphere containing 5% CO_2_. For experiments, RL cells were used in the exponential phase of growth.

Before all experiments, HCT-116 cells were seeded in two-well plates, 150,000 cells/well, and maintained in a 95% humidified atmosphere, 5% (*v*/*v*) CO_2_, at 37 °C overnight. For viability test, SiNPs were then incubated at different concentrations ranging from 0 to 600 µg/mL. After 24, 48, and 72 h of incubation, cell viability was determined by the 3,4,5-dimethylthiazol-2,5 diphenyl tetrazolium bromide (MTT) assay. Thirty microliters of MTT solution (5 mg/mL in PBS) were added to each well and incubation was carried out for 3 h at 37 °C. The culture medium was then removed, and 1 mL dimethyl sulfoxide (DMSO) was added. The absorbance was monitored by a spectrophotometer with a microplate reader at a wavelength of 575 nm. For SiNPs uptake, the same experiments were performed as described for viability. For microscopy observations, the medium was removed and the cells were rinsed twice with phosphate-buffered saline (PBS) with Ca^2+^ and Mg^2+^. For lysosomal labeling, 24 h after SiNPs incubation, an equal volume culture medium of dye working solution of lysosome staining kit (Lysotracker^®^; AAT Bioquest Inc., Sunnyvale, CA, USA) was added for 1 h. The medium was then removed, and cells were rinsed once with PBS for microscopy observations. 

For RL viability, cells were incubated on 96-well-plates at a density of 40,000 cells overnight. Then, particles were incubated at different concentrations (0–100 µg/mL). After 24, 48, and 72 h, cell viability was determined by CellTiter-Glo^®^ Luminescent Cell Viability Assay (Promega, Madison, WI, USA), 100 μL of CellTiter-Glo solution were added to each well, and incubation was carried out for 30 min at 37 °C. The luminescence was monitored by a luminometer with a microplate reader (TRISTAR^2^ LB942, Berthod Technologies). For in vitro labelling of RL cells, 150,000 cells/well were or were not (untreated-0 µg/mL) held overnight with SiNPs at different concentrations. At 24 h, the medium was removed by centrifugation and the cells were rinsed once with PBS for microscopy observations.

### 4.5. Electroporation of Cells

A high-voltage pulse generator electro cell S20 (Leroy Biotech, St Orens, France) was used to deliver pulses of given amplitude U (V/cm), number N, duration T (µs–ms), and frequency F (Hertz). The application of a potential difference U (Volts) across two flat electrodes (parallel 1 cm long, 1 mm thick, and with distance from d = 0.4 cm (or 1 cm)) generated calibrated electric field pulses. Cells in suspension were placed between electrodes brought into contact at the bottom of a petri dish and between which 150,000–500,000 cells (according to the experiment) were placed, suspended in 100 μL of HEPES pulsation buffer (100 mM HEPES, 10 mM MgCl_2_, and 2500 mM sucrose).

Cells were electropermeabilized according to the electric parameters determined by the team for electrogenotherapy (EGT) [28]: 10 pulses lasting 5 ms at a 1 Hz frequency; or the clinically used parameters for electrochemotherapy (ECT) [26]: 8 pulses lasting 100 µs at a 1 Hz frequency. Optimal amplitude was determined to preserve the cell viability.

To determine the cell permeability, cells (500,000 cells) were suspended in 100 μL of pulsation buffer containing 100 μM of propidium iodide (PI, Sigma Aldrich). Cells were then electropermeabilized with various electric field intensities ranging from 0 to 1800 V/cm. Cell suspension was transferred in 300 µL of PBS (GIBCO) without calcium or magnesium to determine the percentage of permeabilized cells by flow cytometry analysis (FACScalibur, Becton Dickson, Franklin Lakes, NJ, USA). 

To determine the cell viability, cells were electropermeabilized as described above and placed in the incubator for 24 h or more. Cell viability was then performed 24 h after EP.

These experiments allowed determination of the optimized amplitude (E (V/cm)) giving the best compromise between viability and cell permeability. The following formula was used to calculate the percentage of permeable and viable cells [44]:

$${\%}_{viable\;cells}+{\%}_{permeabilized\;cells}-100={\%}_{viable\;cells\;permeabilized}.$$

To determine the SiNPs uptake rate, suspended cells were electropermeabilized with optimized EGT or ECT parameters and either put into culture for further analysis or analyzed by flow cytometry to determine the fluorescence intensity of SiNPs in cells.

### 4.6. Microscopic Acquisition and Data Processing

Images were obtained using a Leica DRMIRB fluorescence microscope by using different types of filters: UV (λ_exc_: 340–380 nm, λ_em_: 425 LP) or H3 (λ_exc_: 420–490 nm, λ_em_: 515 nm LP), and L4 (λ_exc_: 460–500 nm, λ_em_: 512–542 nm BP). These filters allowed selective detection of LumiLys 650 NPs (UV or H3) and lysosomal compartments (L4).

Image acquisitions were realized using Metavue software. These acquisitions were then transferred and processed by ImageJ software. Autoscale acquisitions being disabled, it was necessary to process images to reduce saturating colors and make them exploitable. When several acquisitions were made on the same experiment, the intensities of “lightness/brightness” were identical between them in order to be able to correctly study the observed changes.

### 4.7. In Vivo Experiments

Two and a half million RL Cy7 labeled cells were injected subcutaneously (sc) into the right flanks of 8–10 week-old female nude mice, according to the INSERM Animal Care and User Committee-approved protocol (n° 2016-090212091849). Tumor growth was followed using IVIS Spectrum in vivo imaging (Perkin Elmer, Waltham, MA, USA) of fluorescence (at 780 nm).

### 4.8. Statistical Analysis

Statistical analyses were performed with Prism 5 software (GraphPad Software, San Diego, CA, USA). Normality of the samples was verified before using a parametric test (ANOVA, Tuckey) or a non-parametric test (Kruskal–Wallis, Wilcoxon/Mann–Whitney, and a Bonferroni correction). Data were considered statistically significant from a *p*-threshold of less than 0.05 (* *p* < 0.05; ** *p* < 0.01; *** *p* < 0.001).

## 5. Conclusions and Perspectives

Our work provides the evidence that EP allows a more efficient and rapid cell loading with SiNPs. This loading is not limited to the cytoplasm, but also includes the nuclear compartment, while preserving the cell viability. The described labeling procedure is long-lasting, as demonstrated by in vivo fluorescence imaging of labeled tumors. This kind of SiNPs delivery, achieved by EP, could be employed to load extensive amounts of active ingredients, loaded within the core of SiNPs. In this way, active ingredients could be delivered into the cell nucleus, while the particles’ localization could be concomitantly monitored over a period of at least several weeks.

## Supplementary Materials

The following are available online at http://www.mdpi.com/1996-1944/12/1/179/s1, Figure S1: Physical characterization of LumiLys 650 NP. (**A**) LumiLys 650 NPs size distribution measured from TEM micrographs and (**B**) DLS measurements. Figure S2: Spectroscopic characterization of LumiLys 650 NP. (**A**) LumiLys 650 NPs luminescence intensity evolution with various amounts of ruthenium complex. Ruthenium complex amount was expressed as a molar ratio of silicium precursor. (**B**) Emission intensity monitored for 6 h at 650 nm under 365 nm excitation. Figure S3: Spectroscopic characterization of LumiLys SiNPs. Excitation and emission spectra of (**A**) LumiLys 650 and (**B**) LumiLys 780 NPs. Video S1: A time-lapse acquisition of 100 s was performed on HCT-116 cells, incubated with LumiLys 650 NPs and then visualized by wide field fluorescence microscopy (63× magnification). Fluorescence observations were performed before EP, during and just after ECT parameters (8 pulses lasting 100 µs at 700 V/cm). Video S2: A time-lapse acquisition of 100 s was performed on RL cells, incubated with LumiLys 650 NPs and then visualized by wide field fluorescence microscopy (63× magnification). Fluorescence observations were performed before EP, during and just after ECT parameters (8 pulses lasting 100 µs at 800 V/cm).
