# Supplementary material for: Increasing Uptake of Silica Nanoparticles with Electroporation: From Cellular Characterization to Potential Applications
Source: Materials (Basel). 2019 Jan 7;12(1):179. doi: 10.3390/ma12010179 (PMC6337455; doi:10.3390/ma12010179)
Supplement: Supplementary file 1 [file materials-12-00179-s001.pdf]

materials

Supplementary

Increasing Uptake of Silica Nanoparticles with Electroporation: From Cellular Characterization to Potential Applications

Erick Phonesouk [1,2], Séverine Lechevallier [2], Audrey Ferrand [3], Marie-Pierre Rols [1], Christine Bezombes [4], Marc Verelst [2] and Muriel Golzio [1,*]

1 Institut de Pharmacologie et de Biologie Structurale—UMR 5089, 205 route de Narbonne, 31077 Toulouse CEDEX 04, France; Erick_31@hotmail.fr (E.P.); rols@ipbs.fr (M.-P.R.)
2 Chromalys SAS, 29 rue jeanne Marvig, 31400 Toulouse, France; severine.lechevallier@chromalys.fr (S.L.); marc.verelst@chromalys.fr (M.V.)
3 IRSD, Université de Toulouse, INSERM (U1220), INRA, ENVT, UPS, 31024Toulouse cedex 3, France; audrey.ferrand@inserm.fr
4 UMR1037 INSERM, Université de Toulouse, ERL5294 CNRS, 31100 Toulouse, France; christine.bezombes@inserm.fr
* Correspondence: muriel.golzio@ipbs.fr

Received: 10 December 2018; Accepted: 29 December 2018; Published: 7 January 2019

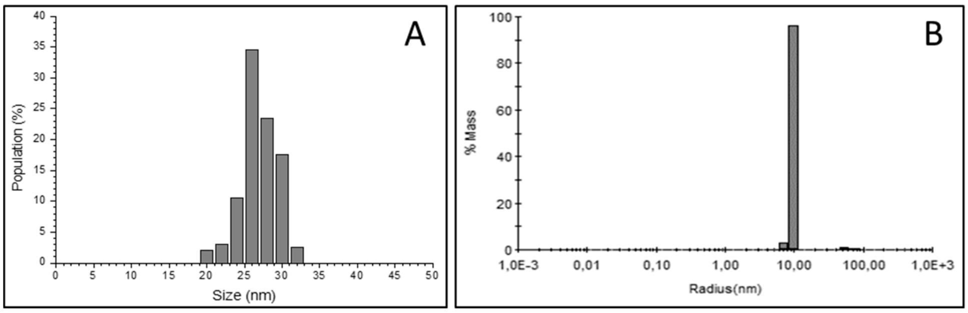

Figure S1. Physical characterization of LumiLys 650 NP. (**A**) LumiLys 650 NPs size distribution measured from TEM micrographs and (**B**) DLS measurements.

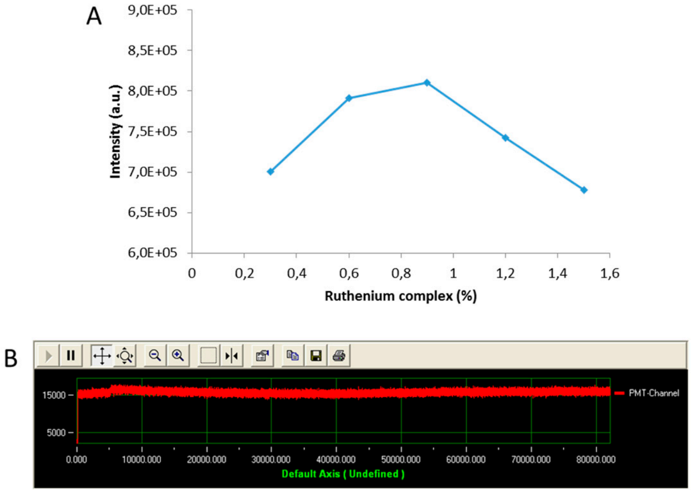

Figure S2. Spectroscopic characterization of LumiLys 650 NP. (**A**) LumiLys 650 NPs luminescence intensity evolution with various amounts of ruthenium complex. Ruthenium complex amount was expressed as a molar ratio of silicium precursor. (**B**) Emission intensity monitored for 6 h at 650 nm under 365 nm excitation.

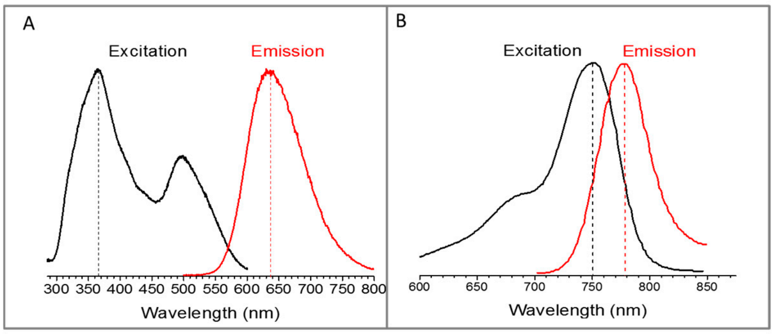

Figure S3. Spectroscopic characterization of LumiLys SiNPs. Excitation and emission spectra of (**A**) LumiLys 650 and (**B**) LumiLys 780 NPs.
